# Clinical Characteristics of Pediatric Clavicular Lesions: A Retrospective Analysis of 20 Cases

**DOI:** 10.7759/cureus.52226

**Published:** 2024-01-13

**Authors:** Bo Jiang, Qian Li, Wang Guo, Li Ju

**Affiliations:** 1 Orthopedics, Children’s Hospital of Nanjing Medical University, Nanjing, CHN; 2 Pediatric Surgery, Children’s Hospital of Nanjing Medical University, Nanjing, CHN

**Keywords:** children, distribution, eosinophilic granuloma, benign bone tumors, clavicular lesions

## Abstract

Background

This research aims to study the diagnostic patterns, anatomical locations, and age-related trends in pediatric clavicular lesions, filling a gap in pediatric-specific data for these conditions.

Methodology

A retrospective study of 20 pediatric patients (aged ≤14 years) with clavicular lesions was conducted based on inclusion and exclusion criteria emphasizing confirmed diagnosis and treatment specifics. The diagnostic process relied on open biopsy, followed by excision or curettage and histopathological examination.

Results

The study primarily involved patients with an average age of 7.1 ± 3.8 years. Eosinophilic granuloma was the most common diagnosis (30% of cases), particularly in the age group of 0-3 years. Clavicular lesions predominantly manifested as either a palpable lump or localized swelling with pain. The medial of the clavicle was the most frequent lesion location. No malignant tumors were found, and the functional outcomes post-treatment were satisfactory.

Conclusions

Pediatric clavicular lesions exhibit distinct diagnostic and anatomical characteristics compared to adults. Eosinophilic granuloma is significantly prevalent in early childhood, necessitating age-specific diagnostic and therapeutic approaches. The study advocates for multidisciplinary collaboration in the treatment and improved understanding of these lesions, which are vital for pediatric orthopedic oncology.

## Introduction

Clavicular lesions in pediatric patients represent a critical yet understudied field within pediatric orthopedics. Though infrequent, these lesions pose considerable diagnostic challenges, often demanding a collaborative, multidisciplinary strategy for precise detection and treatment. Current research provides insights into prevalent pediatric bone lesions [1]; however, detailed investigations specifically targeting pediatric clavicular lesions are notably limited.

The varied anatomical presentation and symptomatology of clavicular lesions add layers of complexity to their diagnosis [2]. Common initial signs, such as a palpable mass or local swelling, typically signal the presence of a clavicular lesion. Nevertheless, the range of underlying pathologies associated with these symptoms is broad, calling for a thorough diagnostic process to ensure correct identification and treatment.

Presently, diagnostic and therapeutic approaches largely derive from research focused on adults [3], resulting in a marked deficiency in pediatric-specific data. This study aims to clarify the diagnostic patterns and precise anatomical locations of clavicular lesions in children. We intend to identify age-related diagnostic trends, assess current surgical management techniques, and map the anatomical occurrence of these lesions within the pediatric population. Through detailed examination and analysis of pediatric cases, this research seeks to fill the existing gaps in knowledge, advocating for a tailored multidisciplinary method in the treatment of pediatric clavicular lesions. Our work seeks to enhance the knowledge in pediatric orthopedic oncology, enhancing patient care and augmenting the general comprehension of clavicular lesions among pediatric groups.

## Materials and methods

Patient selection

A total of 20 pediatric cases were selected based on the following inclusion criteria: (1) patients aged ≤14 years at the time of initial diagnosis; (2) patients presenting with clavicular mass or clavicular pain, with radiological evidence of clavicular lesions; (3) patients with a definitive pathological diagnosis of clavicular lesion; (4) patients who received surgical treatment for clavicular lesions at our hospital from March 2011 to January 2023, with documented ethical approval and consent provided by their legal guardians. Exclusion criteria included (1) patients with osteolytic lesions resulting from post-traumatic osteomyelitis following open clavicular injury; (2) patients presenting with multiple bone lesions including clavicular lesions; (3) patients diagnosed with congenital pseudarthrosis of the clavicle; (4) patients with incomplete medical records.

Initial examination and diagnostic procedures

Laboratory examinations and preoperative radiological investigations were conducted after admission. Routine tests included complete blood count, erythrocyte sedimentation rate, tuberculin skin test, T-cell spot test, and coagulation profile. Radiological assessments aimed at characterizing the osteolytic lesions, lesion structure, and the relationship between soft tissue masses and cortical bone destruction using different imaging modalities. Magnetic resonance imaging and computed tomography were used as further examinations.

Treatment procedures

An open biopsy of the clavicular lesions was performed under general anesthesia. Once malignancy was ruled out, excision or curettage was conducted. In five cases, allogenic cancellous bone grafts were implanted intraoperatively. Postoperative gross specimens were sent for histopathological examination to confirm the diagnosis. After surgery, patients began rehabilitation exercises and regular outpatient follow-up evaluations four weeks postoperatively to monitor healing and functional recovery.

## Results

The study predominantly involved a younger patient cohort (Table 1), with a mean age of 7.1 ± 3.8 years. In terms of gender distribution, males were more prevalent, representing 65% of the cases. Eosinophilic granuloma emerged as a significant diagnosis, being present in 30% of the patients. Additionally, 50% of the grafting procedures were conducted on patients diagnosed with eosinophilic granuloma. Importantly, no malignant tumors were identified. Furthermore, shoulder joint functionality outcomes were excellent, with all patients indicating satisfactory conditions at their final follow-up.

**Table 1:** General information of patients.

ID	Gender	Age (year)	Diagnosis	Side	Symptom	Bone graft	Location
1	Male	5.1	Bone cyst	Left	Mass	Yes	Medial
2	Male	11.8	Enchondromatosis	Right	Incidentally found during chest radiography	Yes	Medial
3	Male	3.8	Enchondromatosis	Left	Mass	Yes	Medial
4	Female	8.6	Osteochondroma	Left	Mass	No	Lateral
5	Male	12	Eosinophilic granuloma	Right	Mass	Yes	Medial
6	Female	9.1	Bone tuberculosis	Right	Mass	No	Medial
7	Female	6.6	Eosinophilic granuloma	Right	Local swelling and pain	No	Medial
8	Female	4.4	Acute osteomyelitis	Right	Local swelling and pain	Yes	Lateral
9	Male	10	Osteochondroma	Left	Mass	No	Lateral
10	Male	5	Eosinophilic granuloma	Left	Local swelling and pain	Yes	Middle
11	Male	4.7	Chondromyxoid fibroma	Right	Local swelling and pain	No	Lateral
12	Male	0.7	Eosinophilic granuloma	Left	Mass	Yes	Lateral
13	Male	8.8	Osteochondroma	Left	Mass	No	Medial
14	Male	11.8	Enchondromatosis	Left	Mass	Yes	Medial
15	Female	10.1	Chronic osteomyelitis	Right	Local swelling and pain	Yes	Lateral
16	Male	10	Eosinophilic granuloma	Right	Mass	No	Middle
17	Male	2.3	Eosinophilic granuloma	Right	Mass	No	Medial
18	Male	5.3	Bone cyst	Right	Mass	No	Medial
19	Female	12.3	Chronic osteomyelitis	Left	Mass	Yes	Medial
20	Female	0.4	Kaporsiform hemangioendothelioma	Right	Local swelling and pain	No	Lateral

**Figure 1:**
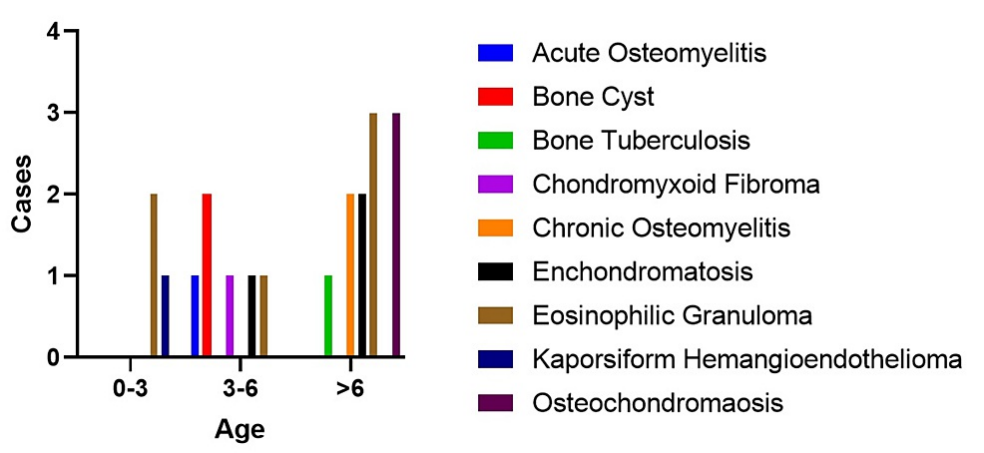
Clavicular lesions in age groups.

In our study, we divided patients into the following three age groups to better understand how certain diagnoses relate to different age ranges: 0-3 years (infants and toddlers), 3-6 years (preschoolers), and over 6 years. Among infants and toddlers, eosinophilic granuloma was the most common diagnosis, accounting for 66.7% of cases. We also exclusively found Kaposiform hemangioendothelioma in this age group. For preschoolers, bone cyst was the most frequently observed diagnosis, accounting for 33.3% of cases. Notably, conditions such as chondromyxoid fibroma, acute osteomyelitis, and bone cyst were only seen in this age group. In the group over six years old, eosinophilic granuloma remained prevalent, representing 27.3% of cases. However, this age group also had cases of osteochondromaosis, enchondromatosis, chronic osteomyelitis, and bone tuberculosis (Figure 1).

**Figure 2:**
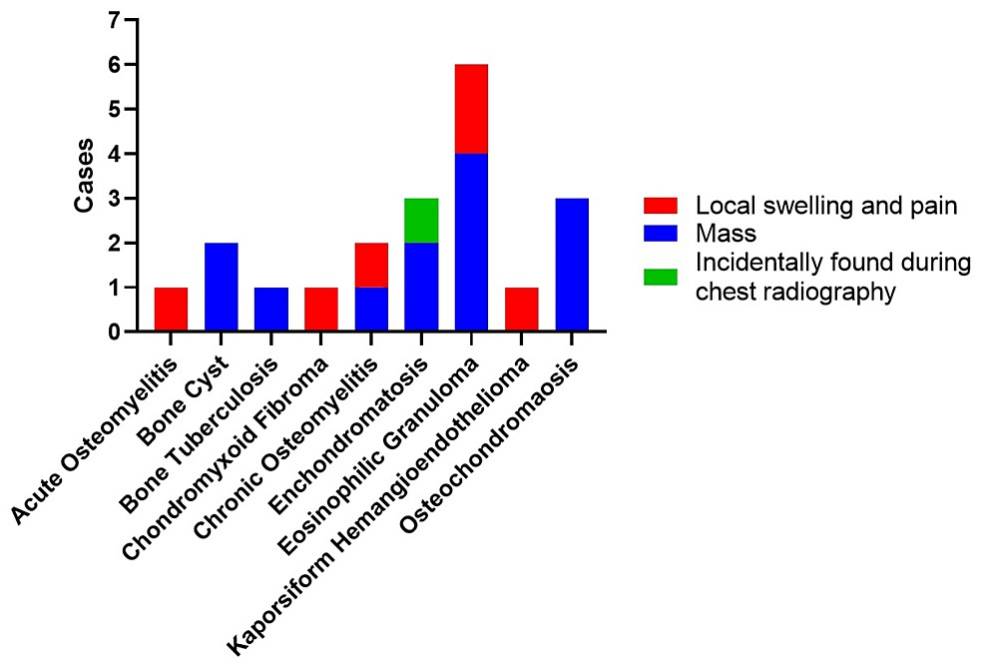
Symptoms of clavicular lesions.

Clavicular lesions manifest mainly in two ways, namely, a palpable lump, often without pain, or localized swelling with pain. When we closely examined the diagnosis, most cases of eosinophilic granuloma had a palpable lump, making up 66.7%, while the other 33.3% had localized swelling and pain. Enchondromatosis had a mixed presentation: 66.7% had a lump, while the rest were found incidentally during chest X-rays. Osteochondroma was always identified by the presence of a lump. On the other hand, conditions such as chondromyxoid fibroma, Kaposiform hemangioendothelioma, and acute osteomyelitis were exclusively associated with localized swelling and pain. In the case of bone tuberculosis, a palpable lump was the only symptom (Figure 2).

**Figure 3:**
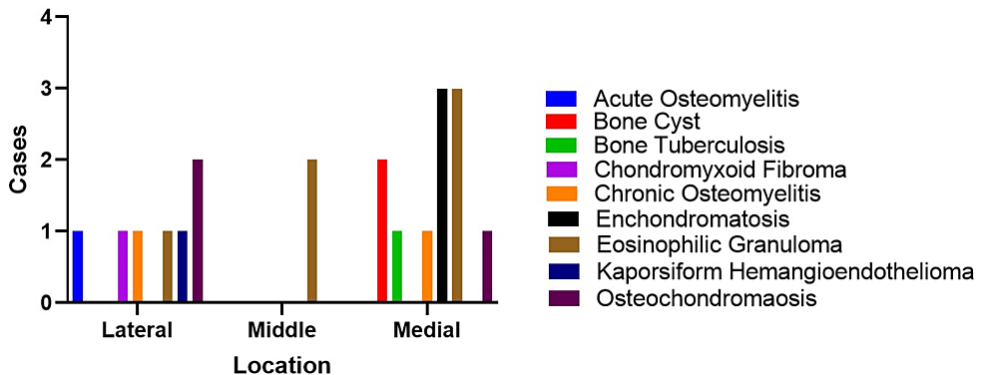
Regions for clavicular lesions.

In this study, we examined where clavicular lesions were located. Patients were categorized based on where their lesions were, i.e., on the lateral, medial, or middle of the clavicle. The results showed that most lesions were found on the medial of the clavicle. Among those with lesions on the lateral of the clavicle, osteochondroma was the most common diagnosis, accounting for 28.6%. Other diagnoses in this area included chondromyxoid fibroma, Kaporsiform hemangioendothelioma, acute osteomyelitis, chronic osteomyelitis, and eosinophilic granuloma, each accounting for 14.3%. On the medial of the clavicle, enchondromatosis and eosinophilic granuloma were the most common, each making up 27.3% of cases. Bone cysts were present in 18.2%, and both bone tuberculosis and chronic osteomyelitis were diagnosed in 9.1% of the medial of the clavicle cases. In the middle region, all lesions were eosinophilic granuloma, representing 100% of cases (Figure 3).

## Discussion

Clavicular lesions in children are uncommon, but it is important to understand where they occur, how they are diagnosed, and what symptoms they cause for accurate treatment. Our study’s findings are in line with existing research on bone lesions in children [4]. The average age in our group was 7.1 ± 3.8 years, which matches what we already know about bone lesions being more common in early childhood, though some benign types may peak slightly later in childhood or adolescence [5]. Eosinophilic granuloma was diagnosed in 30% of patients and was linked to 50% of grafting procedures, highlighting the challenge of managing it. Even though eosinophilic granuloma is not malignant, it can be tricky to treat in kids, especially when it involves the spine, as seen in previous studies [6]. Treatment often requires surgery, such as wide resection with grafting [7].

In this group of patients, we did not find any malignant tumors, which is consistent with what is known, i.e., malignant tumors are less common in children than in adults. However, in rare cases, pediatric clavicular lesions can be malignant, such as Ewing’s sarcoma. There have been reports of Ewing’s sarcoma in the clavicle of children aged 2-15 years, and it was treated with chemotherapy, surgery, and sometimes radiation therapy [8].

This group of pediatric patients showed a variety of clavicular issues across different age ranges. In the 0-3 years group, eosinophilic granuloma was the most common, which matches what we know about eosinophilic granuloma occurring in early childhood [9]. We also found Kaposiform hemangioendothelioma in this age group, which is typically known to appear early in life [10], but its occurrence in the clavicle is a new finding. For patients aged 3-6 years, enchondromatosis was the most common, which is in line with previous research showing it often starts in early childhood due to rapid bone growth during this period [11]. However, the presence of chondromyxoid fibroma and acute osteomyelitis in this age group is somewhat surprising because these conditions are usually seen in older children and young adults [12].

In studying clavicular lesions, we used a precise approach by categorizing patients based on where the lesions were on the lateral, medial, or middle parts of the clavicle. Our main finding showed that the medial of the clavicle was the most common location for these lesions. This differs from previous studies, which found that clavicular tumors were more often on the lateral of the clavicle [13,14]. The difference may be because our study focused solely on children aged under 14, while the other studies primarily centered around adults. Age appears to play a significant role in where clavicular lesions occur.

This study enhances our understanding of clavicular lesions by showing where they are diagnosed. Osteochondroma was most common on the lateral of the clavicle, accounting for 28.6% of cases. On the medial of the clavicle, enchondromatosis and eosinophilic granuloma were both found in 27.3% of cases. Interestingly, the middle part only had eosinophilic granuloma, making up 100% of cases. This matches what we know about eosinophilic granuloma tending to appear in the shafts of long bones, with irregular lytic areas, inner surface erosion, and cortical damage [15]. The presence of eosinophilic granuloma in the middle clavicle region, similar to its tendency in long bones, raises intriguing possibilities for further study. Understanding this distribution may require input from different medical fields, including pediatric orthopedics, oncology, immunology, and genetics.

Subcutaneous lumps or swelling in the clavicle area are usually noticeable and are less likely to be missed by parents or caregivers. However, our research shows that the same underlying condition can present with different symptoms. It may appear as a painless lump that can be felt, or it could come with local tenderness and visible swelling, similar to conditions such as eosinophilic granuloma and enchondromatosis. The only exception is osteochondroma, which usually does not cause pain. Even though these collarbone abnormalities are physically evident, an accurate diagnosis often requires additional testing through biopsy procedures.

The challenge in diagnosing pediatric clavicular lesions arises from the clavicle’s proximity to critical nerves and blood vessels, making needle biopsy a risky procedure. In our study, we preferred an open incisional biopsy, aligning with the cautious approach recommended by Cundy et al. [16]. Their choice came after a case where a delayed diagnosis of clavicle osteosarcoma was linked to a needle biopsy. We opted for this method because pediatric clavicular lesions have a lower likelihood of being malignant, allowing us to obtain a larger tissue sample for accurate diagnosis. Additionally, the open biopsy procedure provides an opportunity during surgery to consider further interventions, striking a balance between precise diagnosis and patient safety.

The study’s sample size is relatively small and may not fully represent the diverse range of pediatric clavicular lesions encountered in broader clinical practice. Additionally, the retrospective nature of this study might limit the scope of data, particularly in capturing long-term outcomes and postoperative complications. Future research with larger, multi-center cohorts and longitudinal follow-up would be beneficial to validate our findings and provide a more comprehensive understanding of these conditions.

## Conclusions

In summary, our study has offered insights into pediatric clavicular lesions, focusing on their diagnostic variations and anatomical patterns. The identification of Kaposiform hemangioendothelioma and the specific distribution of eosinophilic granuloma highlight the complexity of these conditions in younger patients. We believe that a collaborative approach, involving experts from different fields, can be valuable in improving diagnostic procedures and treatment strategies.
